# Revision on Palaearctic species of *Periclistus* Förster with description of a new species and its host plant gall (Hymenoptera, Cynipidae)

**DOI:** 10.3897/zookeys.596.5945

**Published:** 2016-06-07

**Authors:** Juli Pujade-Villar, Yiping Wang, Rui Guo, Xuexin Chen

**Affiliations:** 1 Department of Animal Biology, Barcelona University, Barcelona 08028, Spain; 2 School of Forestry and Biotechnology, Zhejiang A & F University, Lin’an 311300, China; 3 Administration Bureau of Zhejiang Qingliangfeng National Nature Reserve, Lin’an 311300, China; 4 Institute of Insect Sciences, College of Agriculture and Biotechnology, Zhejiang University, Hangzhou 310029, China

**Keywords:** Cynipidae, Gallwasp, inquiline, Periclistus, taxonomy, revision, China

## Abstract

Palaearctic species of *Periclistus* Förster has been systematically described, but a new inquiline gall-wasp, *Periclistus qinghainensis* **sp. n**., is described from China. This species was obtained from an unknown stem gall induced on *Rosa* sp. Diagnosis, distribution and biology of the new species are described in this paper. After examining the types of *Periclistus idoneus* Belizin, 1973 and *Periclistus capillatus* Belizin, 1968, it is concluded that *Periclistus idoneus* belongs to genus *Aulacidea*, and *Periclistus capillatus* is a valid species of *Periclistus*. A key to the Palaearctic *Periclistus* species is also given.

## Introduction

Synergini is an important tribe of the family Cynipidae (Hymenoptera) with a worldwide distribution. They are biologically characterized for being inquilines: although they have lost the ability to induce galls, they are still able to directly modify the gall tissue that surrounds them, inducing the characteristic nutritive tissue usually found in the larval chambers of the gall-inducers (Melika 2006). All inquilines are wholly phytophagous, some of them being lethal if they compete with the inducer for the food in the same larval chamber. This lifestyle represents a unilateral relationship only beneficial for the inquiline (Askew 1984).

The Synergini includes 186 species of inquilines grouped in nine genera (Pénzes et al. 2012^*^). Six genera are inquilines of cynipid galls on Fagaceae (*Agastoroxenia* Nieves-Aldrey & Medianero, 2010, *Ceroptres* Hartig, 1840, *Saphonecrus* Dalla Torre & Kieffer, 1910, *Synergus* Hartig, 1840, *Synophrus* Hartig, 1843 and *Ufo* Melika & Pujade-Villar, 2005); species of *Synophromorpha* (Ashmead, 1903) are also found in *Diastrophus* galls on *Rubus* (Rosaceae); *Rhoophilus* inquilines are found in lepidopteran galls induced by a *Scyrotis* moth (cecidosid) on species of *Rhus* (Anacardiaceae); and inquilines of *Periclistus* Förster are associated with cynipid galls on roses (Diplolepidini).

*Periclistus* is a small genus with 14 species distributed across the Holarctic region, three of them having an uncertain status: *Periclistus idoneus* Belizin, 1973, *Periclistus mongolicus* Belizin, 1973 and *Periclistus capillatus* Belizin, 1968 (Taketani and Yasumatsu 1973).

Despite being morphologically similar to *Synophromorpha* Ashmead, *Periclistus* can be distinguished by the following characters (Ritchie and Shorthouse 1987): uniformly and delicately coriaceous mesoscutum (graniculate or smooth in *Synophromorpha*); notauli never complete, forming two short sulci not posteriorly broadened (complete and distinctly broadened notauli in *Synophromorpha*), ventral margin of subalar triangle with a row of setigerous punctures (without a row of setigerous punctures in *Synophromorpha*), closed radial cells (opened radial cells in *Synophromorpha* and Japanese species of *Periclistus*), and the male’s third flagellomere usually strongly notched and distally broadened (third flagellomere weakly curved, broadly notched and weakly expanded distally in *Synophromorpha*). Both genera form a monophyletic group, as has been demonstrated by several authors (Ritchie and Shorthouse 1987; Ronquist and Liljeblad 2001; Nylander 2004, among others). Here genus *Periclistus* is firstly reported from China, with a new species *Periclistus qinghainensis* sp. n., found in a gall on an unidentifies species of *Rosa* induced by an unknown species in *Diplolepis*.

## Materials and methods

The types of *Periclistus idoneus* and *Periclistus capillatus* described by Belizin from Hurfeish (Israel) and Primorskij Kraj (Russian Far East) respectively, have been examined in this study. They are deposited in ZIN (Zoological Institute of the Russian Academy Sciences, St. Petersburg, Russia).

The galls of the new species described here were collected on May 2010 in the north western province of Qinghai of China. During this month the weather is still cold, the branches of trees are still covered by snow and the useful characters to determine the *Rosa* species are not present in the plant, so it was impossible to identify it; in addition, in China there are approximately 100 described species of *Rosa*, making it hard to establish a potential candidate. Hence, the galls were sent to Y. Wang without determination of *Rosa* species.

The current terminology describing the cynipid gall-wasp morphology follows Liljeblad and Ronquist (1998) and Melika (2006). Abbreviations for the forewing venation are taken from Ronquist and Nordlander (1989) and those for the cuticular surface from Harris (1979). Measurements and abbreviations used here include F1–F12 for first and subsequent flagellomeres. Other abbreviations are: post-ocellar distance (POL), the distance between the inner margins of the posterior ocelli; ocellar–ocular distance (OOL), the distance from the outer edge of the posterior ocellus to the inner margin of the compound eye; and lateral-ocullar distance (LOL), the distance between lateral and frontal ocelli. The width of the forewing radial cell was measured from the margin of the wing to the Rs vein.

Measurements were made under a Leica MZ 12.5 stereomicroscope (Wetzlar, Germany), and photos were taken with a digital camera (Q-Imaging, Micropublisher 3.3 RTV) attached to the Leica MZ APO stereomicroscope (Wetzlar, Germany) using software of Synoptics Auto- Montage version 5.0.

Specimens of the new species are deposited in the Hymenoptera Collection in Zhejiang A & F University
(ZAFU) and the University of Barcelona (UB), respectively.

## Results

### Periclistus capillatus

Taxon classificationAnimaliaHymenopteraCynipidae

Belizin, 1968

Periclistus capillatus Belizin, 1968: 718–719.

#### Type material.

1 ♀ deposited in ZIN, with the following labels: “Kedrovaya pad’ [Nature Reserve] Primorie [= Primorskiy kray] O. Kovalev 17 V 60” (black label, handwritten in Russian), “From galls on leaves of Rosa” (red label, handwritten in Russian), “*Periclistus capillatus* ♀ m. V. Belizin det” (black label, handwritten), “Primorskiy kray, ‘Kedrovaya pad’ ‘Nature Reserve. From galls on Rosa (leaves). 17. V. 60 g. O.V. Kovalev” (black label, handwritten in Russian), “Lectotype ♀ of *Periclistus capillatus* Belizin, 1968, det JP-V 2015” (red label, printed).

#### Diagnosis.

This species is characterized by the following characters: black head and mesosoma, chestnut brown to black metasoma, testaceous antennae and legs; 12-segmented antenna, F1 and F2 subequal in length (4:5); an alutaceous mesoscutum with piliferous points and sparse pubescence; notauli and posterior medial sulcus present, short, both extending to ¼ of total scutum length; parapsidal lines and anterior parallel lines present; smooth mesopleuron; closed radial cell (although both R1 and its projection in margin of forewing nearly inconspicuous), short, 3 times as long as broad; areola visible; metasomal tergites fused (T2+T3) and smooth, with an anterolateral patch of white setae; the subsequent segments are micropunctuated and glabrous.

#### Comments.

This species presents characters belonging to Asian species (scutal and mesopleural sculpture) and characters belonging to European species (radial cell length and shape). A key provided at the end differentiates this species from its congeners.

### Aulacidea idoneus

Taxon classificationAnimaliaHymenopteraCynipidae

(Belizin, 1973) comb. n.

Periclistus idoneus Belizin, 1973: 26.

#### Type material.

1 ♀ deposited in ZIN, with the following labels: Herfeish, 22.IV, Israel, V. Trjapitzin ‘ 966” (black label, handwritten in Russian), “ Holotype *Periclistus idoneus* ♀ m., V. Belizin det” (red label, handwritten), *Aulacidea idoneus* Belizin, 1973, det. JP-V 2015” (white label, printed).

#### Comments.

After examining the holotype, we conclude that this species belongs to genus *Aulacidea*. After determining the specimen following the Palaearctic *Aulacidea* species key made by Melika (2006) we conclude that this species is a valid species related to *Aulacidea laureae* Nieves-Aldrey, 1992 and *Aulacidea follioti* Barbotin, 1972. The three species present the head broader than high, 13-segmented antenna, F1 shorter than F2, incomplete notauli and ciliated forewing margin. *Aulacidea idoneus* differs from *Aulacidea follioti* in presenting median mesoscutal line, like *Aulacidea laureae*; *Aulacidea idoneus* can be distinguished from *Aulacidea laureae* by the following characters: short and narrow scutelar foveae, OOL 3.0 times longer than the diameter of lateral ocellus, space between totuli and clypeus without radiating carina, having shorter notauli and medial mesoscutal line shorter (both extending 1/3 of scutum length) and radial cell (slightly more than 2.0 times longer than broad) and having a second metasomal tergite with only some dorsal points while being laterally smooth.

### Periclistus mongolicus

Taxon classificationAnimaliaHymenopteraCynipidae

Belizin, 1973 species dubia

Periclistus mongolicus Belizin, 1973: 26.

#### Remarks.

This species described from Mongolia was considered by Abe et al. (2007) as having an uncertain status until the types were revised. Because of the loss of the type material (S. Belokobylskij pers. comm.) this species is definitively considered as ‘*species dubia*’ according to the description, which does not permit assessment of its validity nor its placement in the genus *Periclistus*.

### Periclistus qinghainensis sp. n.

Taxon classificationAnimaliaHymenopteraCynipidae

http://zoobank.org/C0EA8F5E-6EAB-4B2F-B77B-1F97332B6066

Figs 1 , 2

#### Diagnosis.

*Periclistus qinghainensis* sp. n. differs from all of the known *Periclistus* species in the absence of notauli. *Periclistus qinghainensis* sp. n. is morphologically similar to two Japanese species (*Periclistus natalis* Taketani & Yasumatsu and *Periclistus quinlani* Taketani & Yasumatsu) and the Far East Russian species (*Periclistus capillatus*) in having a smooth and shiny mesoscutum (very weakly alutaceous in *Periclistus capillatus*) with dispersed piliferous points and smooth mesopleuron, but it differs from all these species in having a partially closed radial cell (radial cell opened in *Periclistus natalis* and *Periclistus quinlani* while closed and shorter in *Periclistus capillatus*), shorter F1 than F2 (F1 and F2 subequal in *Periclistus natalis* and *Periclistus quinlani*) and the absence of notauli (present in the other three species). *Periclistus qinghainensis* sp. n. differs from the European species in having the radial cell partially closed (closed in *Periclistus caninae* (Hartig) and *Periclistus brandtii* Ratzeburg), a smooth and shiny mesoscutellum (uniformly and delicately coriaceous scutellum with a dense and short pilosity without piliferous points in the European species) and the length and width of the radial cell (more than 4.0 times as long as wide in *Periclistus qinghainensis* while around 3.0 times in *Periclistus brandtii* and *Periclistus caninae*).

#### Description.

**Length.** Female. Body length 2.1 mm, and fore wing 2.8 mm.

**Colour.** Body black, except yellow tegulae and antennae, scapus and apical flagellomere darker; coxae dark brown, rest of the legs yellowish; forewing hyaline, with brown veins.

**Figure 1.**
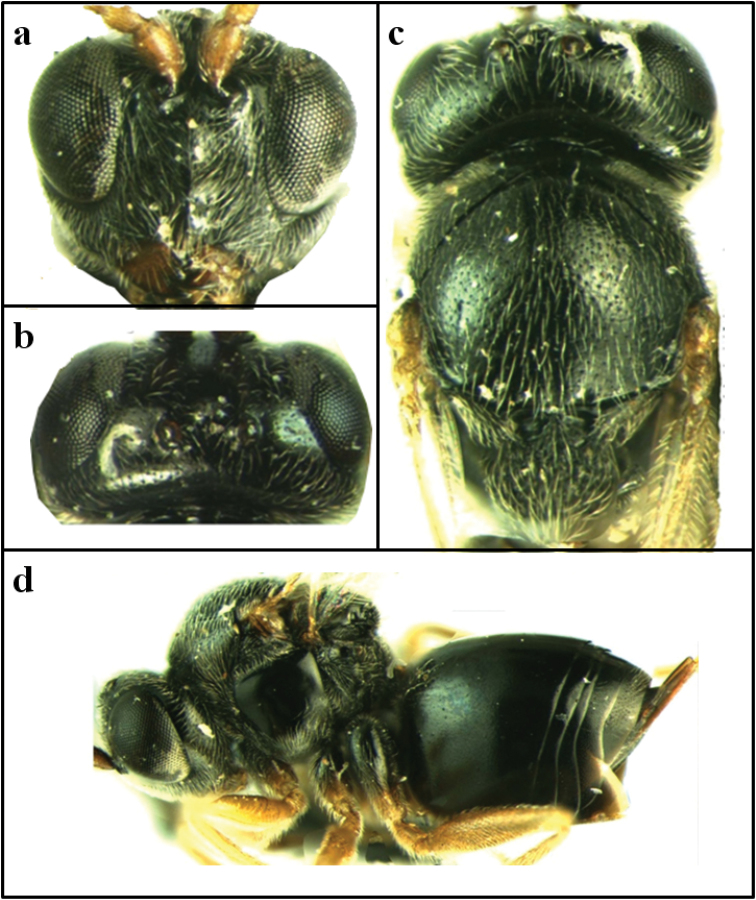
*Periclistus qinghainensis* sp. n.: **a** head of female in front view **b** head of female in dorsal view **c** head and mesoscutum of female in dorsal view **d** general habitus of female in lateral view.

**Head** (Fig. 1a, b). Head coriaceous, with sparse setae, 2.0 times wider than long in dorsal view, 1.4 times wider than high in front view and slightly wider than mesosoma. Gena delicately coriaceous and not broadened behind eyes. Clypeus very small, impressed quadrangular and delicately coriaceous, ventrally slightly rounded; slightly higher than wide, with distinct small anterior tentorial pits, epistomal sulcus and clypeo-pleurostomal lines indistinct. Lower face with striae radiating from clypeus, not reaching eyes and antennal socket, median elevated area delicately coriaceous and striated. Malar space 0.3 times longer than eye height. Diameter of antennal torulus 2.0 times longer than inter-toruli distance and 1.1 times longer than eye-torulus distance. POL: OOL: LOL=1.7: 0.6: 1.3. Frons, vertex, and gena behind eyes and postgena with sparse setae. Frons largely smooth, with some very small and distinct punctures but without lateral frontal carina. Vertex and occiput uniformly punctured.

**Figure 2.**
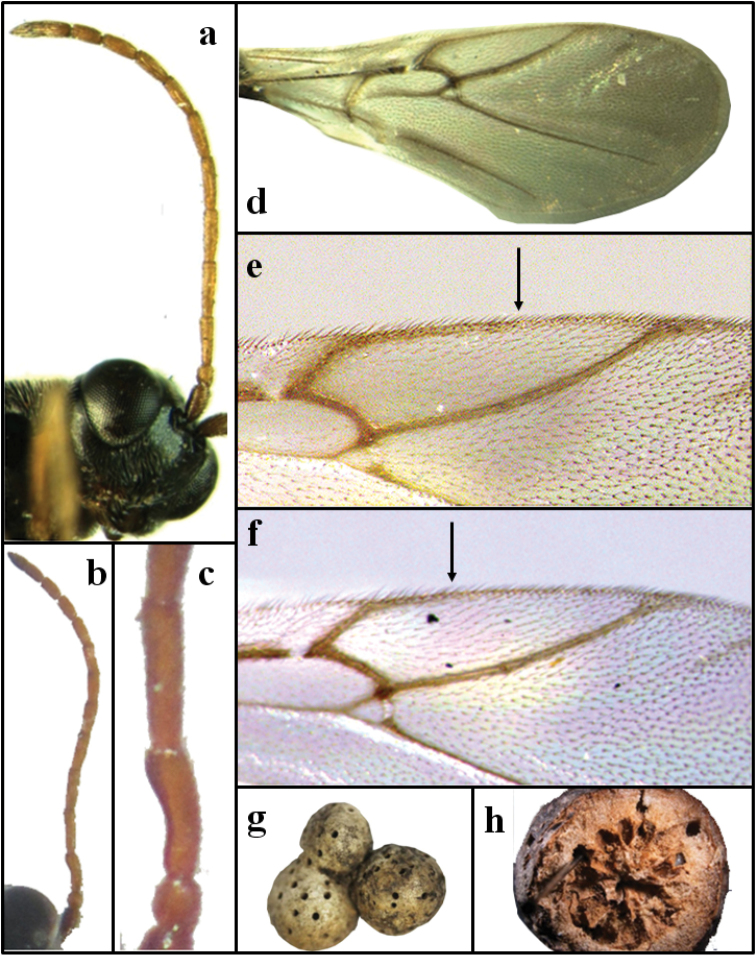
*Periclistus qinghainensis*, sp. n.: **a** antenna and head of female in lateral view **b–c** male antenna and detail of first flagellomeres **d** forewing **e–f** detail of radial cell indicating the R1 prolongation in margin of forewing **g** galls **h** longitudinal section of gall.

**Antenna. Female** (Fig. 2a). 12-segmented, slightly shorter than body; pedicel subglobose, only slightly longer than wide; F1 2.5 times as long as pedicel; F2 around 1.2 times as long as F1 and only slightly longer than F3; the antennal formula is: 9: 4: 10: 13: 11: 11: 10: 9: 9: 8: 7: 14. **Male** (Fig. 2b, c). antenna 14- segmented, F1 medially incised and apically swollen, 2.3 times as long as pedicel, 0.9 times as long as F2; F2 as long as F3; F4 slightly longer than F3; F6–F8 equal in length; F9-10 equal in length; the antennal formula is: 3.0: 2.0: 4.2: 4.9: 5.0: 5.5: 5.0: 4.5: 4.5: 4.5: 4.0: 4.0: 3.5: 4.0.

**Mesosoma** (Fig. 1c, d). Mesosoma slightly compressed dorso-ventrally and longer than high in lateral view, and with white setae. Pronotum dorsal an lateral surface uniformly and delicately coriaceous, lacking wrinkles and lateral pronotal carina but having rounded anterior corners in dorsal view. Mesoscutum smooth and shiny with some dispersed piliferous points, slightly broader than long. Notauli and median mesoscutal line absent; anterior parallel lines distinct, extending to 1/4 of entire mesoscutum length. Parapsidal lines present, shallow, extending to 1/4 of mesoscutum length. Mesoscutum rugose, more sculptured toward central scutellar disk and between scutellar foveae, metanotum slightly overhanging. Scutellar foveae transversely ovate, only slightly wider than high, well-delimited around, with smooth and shiny deep bottom but without setae, separated by distinct medial carina. Mesopleuron smooth and shiny, without striae, with dense setae ventrally, especially postero-ventrally; mesopleural triangle alutaceous, with sparse setae. Metapleural sulcus reaching the mesopleuron at 4/5 of its height; lateral propodeal carinae straight and parallel, with some setae; central propodeal area coriaceous, with setae; lateral propodeal area uniformly and delicately coriaceous, with relatively dense white setae.

**Fore wing** (Fig. 2d–f). Forewing longer than body, wing margin with long cilia; radial cell 4.3 times as long as the wide, partially closed (R1 vein projected about 1/3-1/2 on radial cell margin), Rs and R1 veins slightly curved, areolet distinct; vein Rs+M distinct, nearly reaching basalis.

**Metasoma. Female** (Fig. 1d). metasoma nearly as long as head plus mesosoma, distinctly longer than height in lateral view; metasomal tergites 2+3, with patches of dense setae at laterals in its base, fifth and sixth metasomal tergites broadly punctuate dorso-posteriorly; prominent part of ventral spine of hypopygium very short. **Male.** second and third metasomal tergites not fused, separated by a suture.

#### Type material examined.

Holotype. ♀, China: Qinghai, Huzhu, Bei Mountain (102°32'E, 36°51'N), 2010-V-6, Guo Rui, reared in galls on *Rosa* sp. Paratypes. 6♀♀1 ♂, same labels as the holotype (1♀ paratype UB).

#### Distribution.

China (Qinghai).

#### Biology.

Reared from stem galls on *Rosa* sp. (Fig. 1g and h). The young gall is juicy, soft, covered with small raised tubercles, and multilocular with greenish-purple spots, 1.0-2.0 cm in diameter. Adults emerge in September.

#### Etymology.

The new species is named after the province where it was collected.

## Discussion

**Figure 3.**
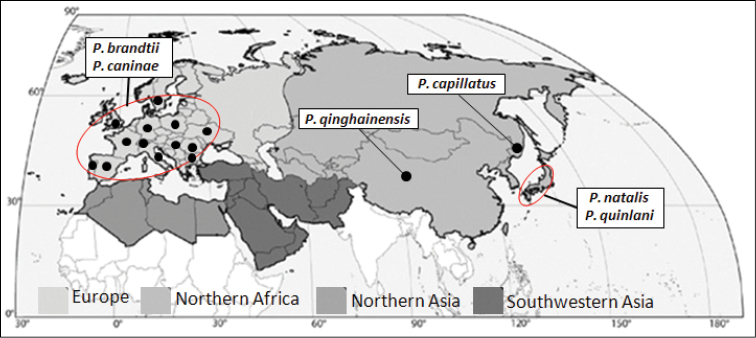
Distribution map of *Periclistus* species in the Palaearctic regions.

*Periclistus* includes 12 species in the Holarctic region, seven species known from America to the north of Mexico (*Periclistus arefactus* McCracken & Egbert; *Periclistus californicus* Ashmead; *Periclistus obliquus* Provancher; *Periclistus piceus* Fullaway; *Periclistus pirata* (Osten Sacken); *Periclistus semipiceus* (Harris); and *Periclistus smilacis* (Ashmead) (Burk 1979; Ritchie and Shorthouse 1987); two (Fig. 3) from the western Palaearctic (*Periclistus brandtii* (Ratzeburg) and *Periclistus caninae* (Hartig)); and three (Fig. 3) from the eastern Palaearctic: *Periclistus capillatus* Belizin from Russian Far East, *Periclistus natalis* Taketani & Yasumatsu and *Periclistus quinlani* Taketani & Yasumatsu from Japan (Belizin 1973; Taketani and Yasumatsu 1973).

*Periclistus* species are associated with *Diplolepis* and *Liebelia* galls, except *Periclistus smilacis*, a Nearctic species known from Florida reared in galls of *Diastrophus smilacis* (Ashmead 1896; Penzes et al. 2012), although M. Buffington and M. Gates (pers. comm.) disagree and consider *Periclistus smilacis* should be associated with some *Diplolepis* species.

Abe et al. (2007) placed *Periclistus capillatus* and *Periclistus mongolicus* in an ‘uncertain status’ and the original description of *Periclistus idoneus* does not allow one to discriminate this species from *Periclistus caninae* and *Periclistus brandtii*, except for the shorter radial cell present in *Periclistus idoneus*. After examining the type material of *Periclistus capillatus* we considered it is a valid species. Unfortunately, the type material of *Periclistus mongolicus* is lost, so we were not able to study it and we considered this species ‘*incertae sedis*’. Finally, when examining the holotype of *Periclistus idoneus* we concluded that it was a valid species belonging to the genus *Aulacidea*.

*Periclistus natalis* and *Periclistus quinlani* are morphologically very similar (both having complete shallow notauli, smooth and shiny mesopleuron, and opened radial cell of the forewing), and share the same gall host (*Diplolepis japonica* (Walker)) and host plant (*Rosa polyantha* Sieb. & Zucc.); however, the authors of these species (Taketani and Jasumatzu 1973) described biological differences between them. Abe (1998) studied the type material of these two species and concluded that there was only one morphological character different between the two species, viz. the pits of the notauli are weakly present anteriorly in *Periclistus natalis*, and absent in *Periclistus quinlani*. Nevertheless, this difference is very superficial based on our knowledge of morphology of Cynipidae; with additional data, it is very probable that both species will be synonymized.

The species described here, *Periclistus qinghainensis*, is similar to two Japanese species (*Periclistus natalis* and *Periclistus quinlani*) and a Far Eastern Russian species (*Periclistus capillatus*). They share a punctured mesoscutum and smooth and shiny mesopleuron. These characters are exclusive of these four species from the rest of the Eastern Palaearctic *Periclistus*. *Periclistus qinghainensis* presents a partially closed radial cell, an intermediate characteristic between the open radial cell of the Japanese species and the remaining of Palaearctic species (*Periclistus caninae* and *Periclistus brandtii* both present a closed radial cell). As mentioned above, *Periclistus capillatus* is intermediate between the Japanese and Chinese species and the remaining of Palaearctic species

### Key to Palaearctic species of *Periclistus*

**Table:** 

1	Mesopleuron entirely smooth, shiny, without striae; mesoscutum smooth or alutaceous, shiny, with sparse setae and piliferous points	**2**
–	Mesopleuron with more or less delicate striae; mesoscutum dull and uniformly coriaceous, with dense setae	**5**
2	Forewing with the radial cell partially closed (Fig. 2e–f); notauli absent (Fig. 1c); metasoma black in females	***Periclistus qinghainensis* sp. n.**
–	Forewing with radial cell opened or closed; notauli shallow but distinct; metasoma reddish-brown in females	**3**
3	Radial cell short, around 3.0 times as long as the width; forewing hyaline	***Periclistus capillatus* Belizin, 1968**
–	Radial cell longer, around 4.0 times as long as the width; forewing with small clouded macula posterior to anterior margin near apex of radial cell	**4**
4	Notaular pits present anteriorly but weakly impressed; and metasoma reddish-brown	***Periclistus natalis* Taketani & Jasumatzu, 1973**
–	Notaular pits absent; and metasoma blackish brown	***Periclistus quinlani* Taketani & Jasumatzu, 1973**
5	Notauli complete; mesopleuron entirely striated, without smooth and shiny patch; fused second and third metasomal tergites of females and third metasomal tergite in males without punctuation or only with some punctures in dorso posterior part	***Periclistus brandtii* Ratzeburg, 1831**
–	Notauli incomplete, absent or very indistinct in the anterior half; mesopleuron mainly striate but with a smooth and shining patch posteriorly; the fused second and third metasomal tergites of females and third metasomal tergite in males with a narrow band of punctuation in posterior part	***Periclistus caninae* (Hartig, 1840)**

## Supplementary Material

XML Treatment for Periclistus capillatus

XML Treatment for Aulacidea idoneus

XML Treatment for Periclistus mongolicus

XML Treatment for Periclistus qinghainensis

## Footnotes

* In the monent to publish this manuscript the Synergini is reestructred according to Ronquist et al. (2015)
